# Use and management of wild fauna by people of the Tehuacán-Cuicatlán Valley and surrounding areas, Mexico

**DOI:** 10.1186/s13002-020-0354-8

**Published:** 2020-01-28

**Authors:** Mariana Zarazúa-Carbajal, Michelle Chávez-Gutiérrez, Yessica Romero-Bautista, Selene Rangel-Landa, Ana Isabel Moreno-Calles, Luis Fernando Alvarado Ramos, Sandra E. Smith, José Blancas, Ek del Val, María del Coro Arizmendi, Alejandro Casas

**Affiliations:** 1 Instituto de Investigaciones en Ecosistemas y Sustentabilidad, IIES, Universidad Nacional Autónoma de México, Antigua Carretera a Pátzcuaro 8701, 58190 Morelia, Michoacán Mexico; 2 Escuela Nacional de Estudios Superiores-Morelia, ENES, Universidad Nacional Autónoma de México, Antigua Carretera a Pátzcuaro 8701, 58190 Morelia, Michoacán Mexico; 3 Centro de Investigación en Biodiversidad y Conservación, CIByC, Universidad Autónoma del Estado de Morelos, Avenida Universidad 1001. Colonia Chamilpa, 62209 Cuernavaca, Morelos Mexico; 4 Facultad de Estudios Superiores-Iztacala, FES-Iztacala, Universidad Nacional Autónoma de México, Avenida de los Baños 1, 54090 Tlalnepantla, Estado de México Mexico; 5 Conservación Biológica y Desarrollo Social A.C., CONBIODES A.C., Calle Nueve núm. 52, Int. 4, Colonia Espartaco, Coyoacán, 04870 Ciudad de México, México; 6 Posgrado en Ciencias Biológicas, Unidad de Posgrado, Universidad Nacional Autónoma de México, Edificio A, 1° Piso, Circuito de Posgrados, Ciudad Universitaria, Coyoacán, 04510 Ciudad de México, Mexico

**Keywords:** Animal management, Biodiversity conservation, Domestication, Ethnozoology, Mesoamerican ethnobiology, Tehuacán-Cuicatlán Biosphere Reserve

## Abstract

**Background:**

Interactions between humans and fauna lay in the heart of the history of human subsistence. In Mesoamerica, the Tehuacán-Cuicatlán Valley (TCV) harbours a high biodiversity with archaeological and ethnoecological evidence of its use by people inhabiting the area since at least 12,000 B.P. It is recognized as one of the most ancient areas of agriculture in the Americas, and a broad spectrum of management practices aimed to ensure the availability of desirable plants has been documented, but it has not been analysed for animals. This study aimed to investigate the use and management practices directed to wild animals along current settlements within the TCV and neighbouring areas.

**Methods:**

We conducted an extensive search, review and analysis of documental sources for the period between 1967 and 2018. We found 38 documents providing information about the presence of animal species and 15 describing their use and/or management. We included our own observations from four case studies among the Ixcatec, Cuicatec, Nahua and Mestizo people, as well as from regional studies of biodiversity. We used unconstrained multivariate data analysis to describe the management typology of the animals in the region.

**Results:**

Hitherto, 652 vertebrate species and 765 species of insects have been recorded in this area; and until present, 107 wild animal species have been reported to be used in 11 use-type categories, mostly for food (65.42%), ornamental (27.52%) and medicinal (21.10%) purposes by the Nahua, Cuicatec, Popolocan, Ixcatec, Mazatec and Mestizo people. Their extraction entails manual capture and gathering as well as hunting and trapping strategies, some of them involving planning in time or space and communitarian regulations; in addition, relocation actions and care in captivity were recorded. Nearly 178 of the species distributed in the region with no reports of local use are used in other localities of Mesoamerica. Ethnozoological information is still lacking for the Mixtec, Chinantec and Chocholtec people in the area.

**Conclusions:**

Wild fauna is still a valuable resource for the inhabitants of the TCV. Animals are obtained through extractive practices, which vary from one another in their qualitative attributes. With this work, we provide a context for further research priorities on fauna management in a region of high biocultural significance.

## Background

Animals have been part of human life since its origins, representing sources of food, medicine, clothing, social, cultural, symbolic and spiritual benefits for humanity [1]. Human groups integrate faunal elements to their subsistence systems through a broad range of practices [2], some of them, on one hand, involving control of the reproduction of successive generations and artificial selection processes of some animals that result in their domestication. On the other hand, people’s practices might include the opportunistic extraction of animals through hunting or gathering. However, people may also take deliberate actions to ensure the availability of wild fauna, without necessarily involving a domestication process [2]. In order to improve the availability of desirable animals, these management actions might involve different ecological scales, from individuals to whole biotic communities [3] or even ecosystem processes.

Management practices include multi-scale decision-making and transformations in order to use, maintain or recover elements or functional processes of ecosystems [4]. In the case of fauna, these strategies can be focused on the animals themselves, from the individual to the population level, but also on elements or processes that support or influence them [2], such as their ecological mutualists (i.e. host plants, plants of their diet, plants pollinated and/or dispersed by them), preys and/or predators, and even manipulation of abiotic elements like water and fire [5]. The set of management practices can vary in the amount of energy and human effort invested, the presence of artificial selection, the control of the reproductive systems, and the extent of abiotic elements used, among others [6]. This variation can be pictured along a gradient of management “complexity” and/or “intensity” [4, 6].

The kind and intensity of management practices directed to animals depend on the motivations and the nature of the interactions between humans and fauna. Such interactions are modulated by both individual and social experiences and knowledge that are part of the complex of knowledge “corpus”, beliefs “cosmos*”* and practices “praxis” known as traditional ecological knowledge or TEK [7, 8]. In Mesoamerica, a region recognized for its high biocultural richness [9, 10], and as one of the world’s main centres of domestication and origins of agriculture [11], the wide spectrum of human-fauna interactions has configured a variety of processes and management practices. These, however, have not included the domestication of large mammals (i.e. for meat, milk or textile fibre production as well as draught animals) as in the Middle East, some other regions of Eurasia and the Andean Region [12].

The Mesoamerican spectrum of human-fauna interactions has included management strategies that resulted in the post-domestication selection of the dogs (*Canis lupus familiaris* Linnaeus, 1758) that arrived at the continent with human immigrations [12] and the domestication of the turkey (*Meleagris gallopavo* Linnaeus, 1758) [13]. However, a variety of animals have received human attention in several ways. One example is the systematic nurturing of insects (i.e. *Dactylopius coccus* Costa, 1829*, Melipona* spp. Illiger, 1906) [14, 15] and vertebrates (i.e. *Ara militaris* Linnaeus, 1766, *Sylvilagus* spp. Gray, 1987) [16–18]. Currently, along with the nurturing of these species and the adoption of introduced domesticates from all over the world (i.e. *Apis mellifera* Linnaeus, 1758, *Capra hircus* Linnaeus, 1758, *Bos taurus* Linnaeus, 1758, *B. taurus indicus* Linnaeus,1758, *Ovis aries* Linnaeus, 1758, *Equus caballus* Linnaeus, 1758, *Gallus gallus* Linnaeus, 1758, *Sus scrofa scrofa* Linnaeus, 1758, *Cairina moschata* Linnaeus, 1758 and other species of Anatidae), there is a range of extractive practices of wild animals. Some of them involve temporal captivity of individuals without breeding [19, 20], the active cultivation of “milpa” exclusively for prey attraction [3], favouring and caring for host plants in situ to increase the amount of insects gathered [21], the care on structures such as ant nests to maintain their availability [22], the spatial and temporal planning (i.e. hunting, fishing or gathering closures) to obtain a given resource and the formulation of communal and even religious regulations [23, 24], among others. All these strategies are intended to satisfy several human concerns.

Human needs for food and medicine are the main drivers of management, but in addition, factors like curiosity, beauty and empathy can also be relevant [20, 25]. Furthermore, actions are taken to reduce or prevent damages or losses that faunal elements may produce for subsistence systems or human health [26, 27]. Ethnobotanical evidence supports the hypotheses that management practices are primarily directed to increase the availability of those resources under a high demand pressure due to their high value for use, especially when resources are relatively scarce [6, 28–30]. However, the reduction in the risk of extirpation of a given resource after management might not always be successful, causing the loss not only of a biotic resource but also of the social and cultural activities related to it [30].

The analysis of the balance between the demand and the outcome of management practices to improve or maintain the availability of desirable animals is in the interest of developing the theory of integrated ecosystem management. With this work, we wanted to contribute with a first regional analysis of the contemporary use and management practices of fauna (i.e. excluding zootechnical methods used to the intensive breeding and exploitation of animals and their products, especially for the introduced varieties of animal domesticates from all over the world) by the indigenous and mestizo people within a region of Mesoamerica with high biocultural diversity, in which archaeological and ethnoecological evidence indicates the use and management of plants and animals for at least 12,000 years [4].

We aim to determine the number of species of wild animals that are used, and describe how they are used, the needs they satisfy, and the management practices involved in obtaining, maintaining or restoring the animal populations along a gradient of management intensity, in the Tehuacán-Cuicatlán Valley and neighbouring areas. We expected to find that wild animals continue being important elements of people’s subsistence system in the region, that vertebrates are mainly used as food, medicine or pets [1], that edible insects are mostly represented by Coleoptera, followed by Hymenoptera, Hemiptera, Orthoptera and Lepidoptera, following the general tendency reported for Mexico [31–33]. Finally, we expected to find a variety of management practices devoted to wild fauna including reproduction in captivity, captive care without reproduction, protection of animals or their host plants in wild conditions, hunting and gathering.

## Methods

### Study area

**Fig. 1:**
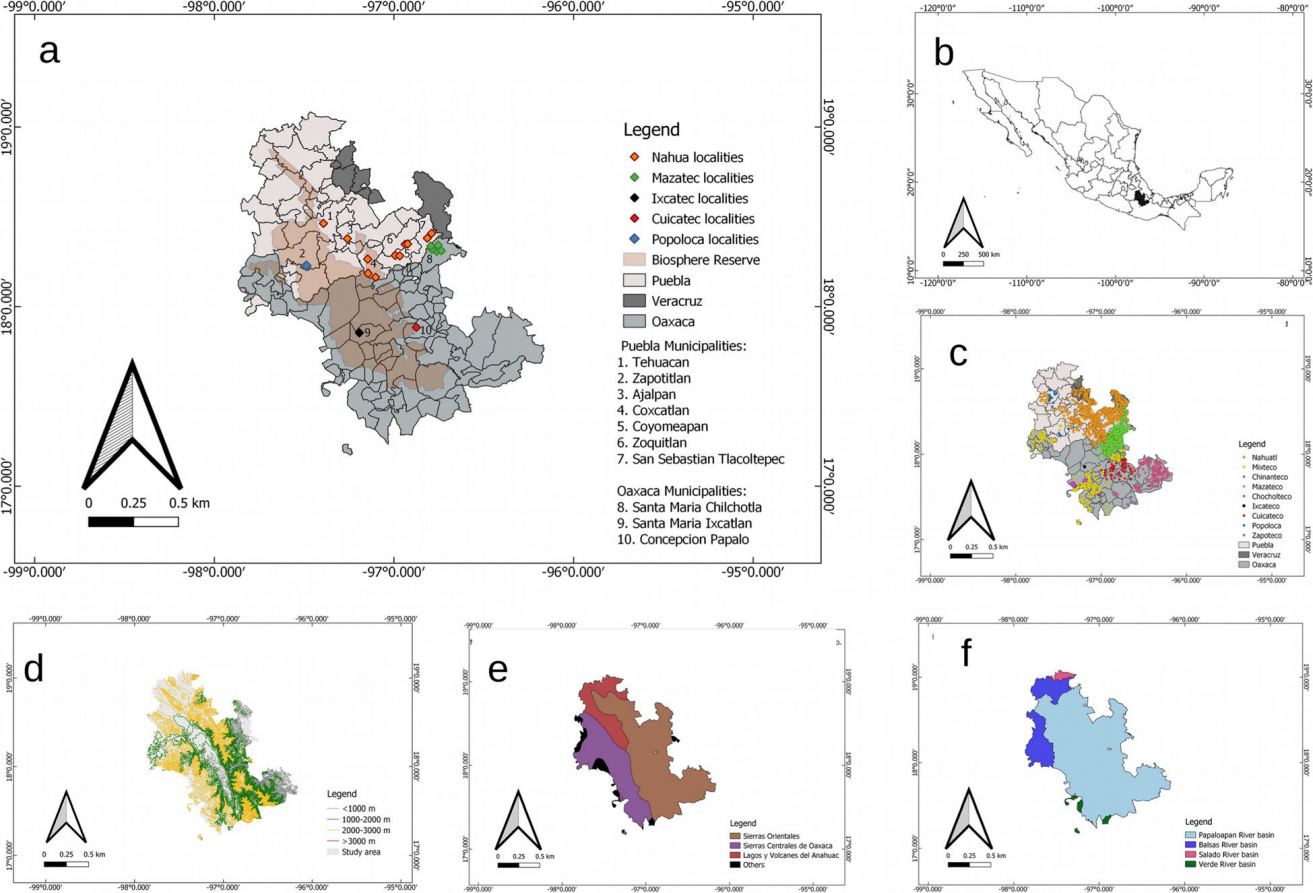
Study area. **a** Political division of the study area in the Tehuacán-Cuicatlán Valley and surrounding regions showing indigenous localities with use or management of fauna records and the Tehuacán-Cuicatlán Biosphere Reserve. **b** Study area in the states of Puebla and Oaxaca, in central Mexico. **c** Distribution of indigenous languages in the localities within the study area following Ávila-Blomberg et al. [36]. **d** Elevation gradient in the study area. **e** Main physiographic regions within the study area following Cervantes-Zamora et al. [37] **f** Hydrological basins within the study area. The figures were elaborated in Qgis 3.8 [38] with data available from the website Geoportal CONABIO [36, 37, 39–41]

The Tehuacán-Cuicatlán Valley (TCV) is an area of about 10,000 km^2^, located in south-eastern Puebla, north-western Oaxaca and the limits of Veracruz [34, 35] (Fig. 1). A high environmental heterogeneity can be found in the area, with warm, semi-warm and temperate climates and average annual precipitation ranging from 400 to 700 mm [42]. The region is part of the Papaloapan River basin and comprises several valleys and mountain chains with elevations from 595 to 2950 m above the sea level [43]. The surrounding mountains, belonging to the physiographic regions known as “Sierras Orientales” and “Sierras Centrales de Oaxaca”, harbour a gradient from thorn-scrub, tropical deciduous forest, oak forest, pine-oak forest in leeward slopes, and cloud forest and tropical rain forest in windward slopes [35]. The orographic shadow caused by the Sierra Madre Oriental prevents humidity from the Gulf of México and causes the arid condition of the TCV. The TCV is one of the five floristic provinces belonging to the Mexican Xerophytic Region in the Neotropical region [44]. At least 29 plant associations belonging to six general vegetation types have been recognized in this area, which stands out from other arid zones in North America for its high floristic diversity [34, 43] and its high species richness of mammals and birds [45].

The use and management of regional biodiversity have been part of the subsistence strategies of the human groups inhabiting the TCV for approximately, the last 14,000 years [46]. Wild plants and animals ranging from big vertebrates (i.e. antelopes, horses, deer) to insects have been part of the human diet since prehistoric times [47, 48]. Archaeological records suggest that they were obtained through hunting, trapping and gathering [47]. The management of agroforestry systems, a current practice in the area [49, 50] is also millenary in the region [46]. The human groups currently inhabiting the Valley (i.e Ixcatec, Nahua, Cuicatec, Mixtec, Popolocan, Chocholtec, Chinantec, Mazatec and Mestizo) use over 2,000 of the more than 3,000 vascular plant species occurring in the TCV, and at least 610 of them are subject to management practices [4, 25]. However, information regarding the current use and management of fauna by human groups in the area is not systematized. In 1998, a Biosphere Reserve of about 500,000 ha was decreed by the Mexican Federal Government Ministries SEMARNAT and CONANP [51] within the TCV. Within this Biosphere Reserve, an area of 145,255 ha was included in 2018 as a Mixed Site in the World Heritage List by the UNESCO because of its cultural and biological significance [52].

In our research, we included documented contemporary records of fauna occurring in any of the 107 municipalities (1436 localities) of the TCV and surrounding areas (Fig. 1a; the list of localities and municipalities is provided in the Additional file 1). We also included evidence from the archaeological records analysed by Kent V. Flannery, Eric O. Callen and Richard S. MacNeish [46–48].

### Documental and field research

We conducted documental research between January 2018 and January 2019 for the available scientific literature about the occurrence, use and management of wild fauna in the studied region. Firstly, we consulted key documents (published between 1967 and 2018 according to the taxa) [47, 48, 51, 53–71]. Afterwards, we consulted the digital repositories of EBSCO, SCOPUS, Web of Knowledge and Google Scholar for subsequent records; in addition, those of specialized journals (Therya, Revista Mexicana de Biodiversidad, Acta Zoológica Mexicana, Etnobiología, Huitzil, Journal of Insects for Food and Feed, Journal of Ethnobiology and Ethnomedicine, Journal of Ethnobiology, Ethnobiology letters, Ethnobiology and Conservation); and those from dissertations of three Mexican universities (UNAM, UAM, BUAP). Our search included 70 keywords in English and Spanish (Additional file 2). In order to collect the information, we considered the following criteria: (a) geographic: we included those records of the occurrence, use or management of fauna that felt within the study area (see Fig. 1 and text in the “Study area” section), (b) conceptual: for management, we took Zeder’s approach and excluded works about zootechnical intensive management (see “Background” section), (c) time-line: all included records are subsequent to the research by Flannery, Callen and MacNeish 1967, (d) publication criteria: theses, dissertations, peer-reviewed journals, published abstracts and, in the case of fauna occurrence records, we included 7 databases hosted by CONABIO and GBIF). These criteria made possible to include practically all found works except for two sources that felt out of the geographical area and were instead considered in the references as other areas of Mesoamerica. We included records about the presence of fauna from 38 documentary sources (21 scientific reports published in peer-reviewed journals, seven public databases, four lists, three book chapters, two dissertations, one book) and registers about the use or management of this fauna from 15 documentary sources (five scientific reports published in peer-reviewed journals, four dissertations, three book chapters, one book, one scientific dissemination article, one conference abstract). We also included primary information generated through the inventory of records of our own observations of living individuals, hunting evidence, and commercialization and interchange in the regional markets, as well as mentions in semi-structured interviews conducted with 30 Cuicatec households [72], 30 people in a mestizo locality with Popolocan ancestors in Zapotitlan, Puebla [73], 16 Ixcatec people in 2016, and with 16 people in three Nahua localities in Coyomeapan, Puebla (2018-2019). Additionally, we went through the checklist of species present in TCV with no local reports of use and checked their uses reported in other regions of Mesoamerica. This search based on the set of animal species whose presence has been reported so far within the TCV (references cited in Table 1). Specifically, if ethnozoological information for an animal species occurring in the study area but no information reported for the TCV region, we searched for at least one kind of use of the animal outside the TCV. This was done in order to analyse the supposition that in the TCV region, there would be more species under any use or management categories than those that have been documented so far. We included 38 documental sources (25 scientific reports published in peer-reviewed articles, ten book chapters, one dissertation, one book, one scientific dissemination article). To avoid repeated information associated with synonymy, the nomenclature was checked with the Integrated Taxonomic Information System 2019 and 2000 [144].

**Table 1:** Number of animal species reported for the study area and the proportion of species used in the region and in other parts of Mesoamerica

Taxonomic group	P: Number of species present in the VTC^a^	Number of Orders, Families, Genera, Species (UR)^b^ with current use report in the VTC	Number of Orders, Families, Genera, Species (URO)^c^ with use report in other regions	Species with use reports in the TCV (UR/P*100) (%)	Species with use reports in the TCV and other regions ((UR+URO)/P)*100) (%)
Vertebrates	652	25, 51, 74, 75	25, 60, 121, 158	11.50	35.73
Aves	372	13, 26, 39, 41	16, 37, 81, 107	11.02	39.78
Mammalia	133	9, 17, 24, 27	4, 7, 13, 16	20.30	32.33
Reptilia	93	2, 7,10, 7	3, 10, 20, 26	7.52	35.48
Amphibia	38	0	2, 6, 7, 9	0.00	23.68
Actinopterigia	16	1, 1, 1, 2 Nd	Nd	1.25	Nd
Insecta	765	5, 19, 33, 32	4, 9, 16, 20	4.18	6.79
Lepidoptera	368	–,7, 11, 11	–, 4, 11, 15	2.98	7.06
Hymenoptera (Apidae)	262	–, –, 2, 2	–, –, 1, 1, 1	0.01	1.14
Hymenoptera (Vespidae)	Nd*	–, –, 4, 4	Nd	Nd	Nd
Hymenoptera (Formicidae)	31	–, –, 2, 3	–,1,1,1	9.67	12.90
Coleoptera	72	–, 5, 5, 5	–, 3, 3, 3	6.94	11.11
Hemiptera	24	–, 3, 5, 5	Nd	20.83	Nd
Orthoptera	8	–, 1, 4, 4	Nd	50	Nd

Nd*: Not determined. a,b,c: References: ^a^Mammalia: [56–58, 74–81]; Reptilia and Amphibia: [47, 54, 59, 82–87]; Aves: [47, 51, 62, 73, 88–93]; Actinopterigia: [94]; Lepidoptera: [63]; Apidae: [64, 65, 95]; Formicidae: [66]; Coleoptera: [67, 68]; Hemiptera: [69, 96]; Orthoptera: [70]. List of species is not provided but see “References” section for the consulted literature)

^b^[33, 47, 51, 72, 73, 97–106], Rangel-Landa, Smith, Zarazúa and Chávez, this study. See Additional file 3 for a summarized list of the species and Additional file 4 to see the full records (in Spanish)

^c^[31–33, 107–143]. See Additional file 5 for the list of species (in Spanish)

### Data analysis

#### Faunistic reports since 1967

We calculated the proportion of used species per taxonomic Class, in relation to the total number of species of those classes recorded in the TCV since 1967, the date of publication of the studies by Kent V. Flannery in The Prehistory of the Tehuacan Valley [47]. We also estimated the proportion of species occurring in the TCV that, although not used in this area, are used somewhere else in Mesoamerica according to our literature review.

We grouped the reported uses in eleven categories: (1) edible: the animals that provide food, (2) ornamental: those used as decoration because of their beauty while alive or prepared after death, (3) medicinal: animals providing ingredients to be used in the attention of human health or childbirth, (4) weather indicator: cases in which the presence of an animal species or its phenotype is used as indicator of rain, temperature or seasonal changes, (5) ceremonial: animals used for ritual celebrations or religious purposes, (6) animal companion or pets: those required as companionship, beyond decoration or edible uses, (7) amulet: animals or their parts that bring good fortune and protection, (8) recreational: animals implied in human leisure with the exception of pets; an example is birdwatching, (9) tool: parts of the animal (for instance bones) that are used to facilitate a mechanical work, (10) crop protection: an animal, its parts or its secretions employed to protect cultivated plants against damages and (11) melliferous: animals producing honey consumed by humans. As an indicator of each species’ relevance, we summed the number of use categories in which each species was recorded [145]. For medicinal fauna, we recorded the number of species used for each illness reported. Trochilidae Vigors, 1825 and Poeciliidae Bonaparte 1831 were analysed as Families while *Sylvilagus* spp. and *Sciurus* spp. Linnaeus, 1758 as Genera because their identification to a lower level is often not reported or has not been confirmed. Descriptive statistics were used to analyse information for all and each taxonomic Class.

Management practices were grouped into seven general categories: (1) manual capture and gathering: the manual collection of vertebrates or invertebrates, (2) hunting: the unplanned or minimally planned chasing and killing of animals, (3) care in captivity: maintaining and feeding animals in human areas while restricting animal movement capability, (4) planned gathering/hunting: these activities when involving the design of an agenda in time and space to capture the animals, the organization of participants and, sometimes, the establishing or attending of previously established agreements and restrictions, (5) enhancing: acts aimed to increase the presence of animals in situ, (6) relocation: transporting the animals into the desired place, in order to increase their numbers or to facilitate their collection or that of their products, and (7) trapping: the use of handmade cages or traps to capture animals. As an indicator of the variety of management forms for each species, we summed the number of management categories in which each species was recorded [145]. Basic descriptive statistics were calculated overall and for each taxonomic Class.

We recorded the vegetation types in which used fauna has been reported to occur in the TCV, following Valiente-Banuet et al. [43]. We referred to the six main vegetation types identified by these authors (i.e. A-F) as: A: columnar cacti forest, B: lowland tropical deciduous forests (< 1800 m), C: highland temperate (pine-oak) forests (> 1800 m), D: riparian vegetation, E: xerophytic shrubland, F: shrubland. In accordance with our records, we also included cloud forest, tropical rain forest and sub-deciduous forest (see Table 1 for sources). If a species occurred in several vegetation types, we considered each of them.

Unconstrained principal component analyses (PCA) were performed to explore the variation of the used animal species (*n* = 71, the complete checklist of species is provided in Additional file 3) according to three variables: (1) the number of use categories, (2) the number of management categories and (3) the number of cultural groups that use/manage the animal in the TCV region. In order to detect correlation among these numerical discrete variables, we performed Spearman correlation tests.

To analyse how the contribution of the animal management actions influences variation in the management typology, we performed an unconstrained PCA based on a presence/absence matrix. For constructing the presence/absence matrix, we included the species as rows (*n* = 47), and the following ten variables as columns: (1) use of fire guns, (2) hunting in the milpa, (3) use of dogs, (4) use of traps, (5) manual capture and opportunistic gathering, (6) feeding in the wild and use of baits for attraction, (7) captivity, (8) planned gathering of individuals including communitarian regulations, (9) extraction of structures and (10) relocation of animals or structures. We excluded those species with no detailed information about management actions beyond a general category. PCA analyses were performed with the ‘prcomp’ function of the ‘stats’ package and the results were visualised with the ‘factoextra’ package of the R software [146, 147].

#### Faunistic reports between 12,000 B.P and 500 B.P.

We integrated a list of the animal species used and managed in the human subsistence systems of the TCV between 12,000 B.P. and 500 B.P. based on archaeological findings [46–48]. In this list, we included vertebrate species that were identified to be part of the human diet through signs of manipulation in bones, the age structure of the animal remains (i.e. deer, cottontail), and traces of projectiles and traps [47]. We, in addition, included insects inferred to be part of the diet based on the content of human coprolites [48], but we excluded coprolites containing remains of bones or hair to reduce the chances of reporting the diet of non-human mammals. We complemented the list with the type of uses mentioned in the ethnozoological research from 1967 [47] to 2019.

## Results

### Use of the regional fauna

Vertebrates in the TCV were represented by 652 species, nearly 11.50% of them reported as being used by people of the area. However, when including the uses reported for other regions of Mesoamerica, we found that 35.73% of the vertebrate species occurring in the TCV are potentially used as resources. Out of the 765 species of insects that have been reported in the TCV, only 4.18% was reported to be used in the region, but about 6.79% of them are used in other regions of Mesoamerica (Table 1).

**Fig. 2:**
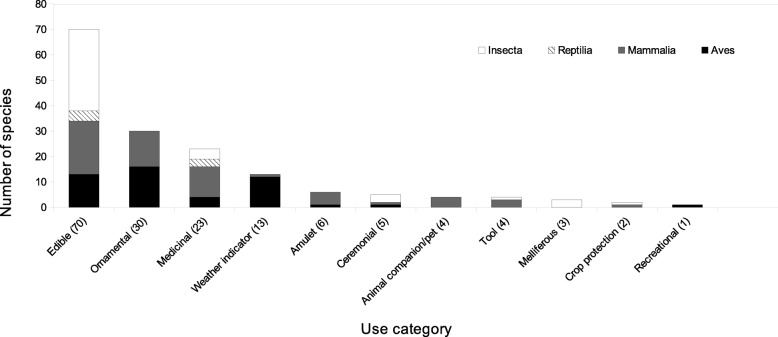
Use categories for animals in the Tehuacán-Cuicatlán Valley and surrounding regions. Totals are indicated in parentheses

Overall, the animals used in the TCV were found distributed in the following use categories: 70 edible species, 30 ornamental, 23 medicinal, 13 weather indicators; six are used as amulets, five as ceremonial or for rituals, four as animal companion and tools, respectively, three are melliferous, two are used for crop protection and one for recreational purposes (Table 2). The number of different uses per species ranges from 1 to 5 (all species mean = 1.54 ± 0.08 SE, *n* = 109; birds mean = 1.36 ± 0.09 SE, *n* = 36; mammals mean = 2.34 ± 0.22 SE, *n* = 26; reptiles mean = 1.14 ± 0.14 SE, *n* = 7; insects mean = 1.28 ± 0.12 SE, *n* = 38). The species with more uses reported are the naturalized bee *Apis mellifera* and the temazate deer *Mazama temama* Kerr, 1792 (Fig. 2)*.* Bee’s larvae are edible, honey is extracted, wax is used as glue, to elaborate candles with ceremonial motives and to make art pieces. The meat of the temazate deer is edible, the male reproductive structures are used to facilitate childbirth, heads and legs are prepared through taxidermic techniques to be used as ornaments and their skulls are exposed as a protection against “mal aire” (cultural illness or the agent that causes it).

**Table 2:** Number of animal species per use and management category, in the TCV and surrounding regions

	Use categories^a^											Management categories^b^						
Number of species	E	O	M	WI	C	R	AC	A	T	CP	ME	C/G	H	CC	PG	E	R	T
Aves (41)	13	16	4	12	1	1		1				14	13	13		3		2
Mammalia (27)	21	14	12	1	1		4	5	3	1		6	19	6				
Reptilia (7)	4		3									3	3					
Lepidoptera (11)	11		1									5			3		1	
Hymenoptera Apidae (2)	2	1			1				1		3	3						
Hymenoptera Vespidae (4)	4				2							3						
Hymenoptera Formicidae (3)	3		1							1		2			1		1	
Coleoptera (5)	3		1															
Hemiptera (5)	5														3			
Orthoptera (4)	4											1						
Total	70	30	23	13	5	1	4	6	4	2	3	37	35	19	7	3	2	2

^a^Use categories: *E*: Edible, *O*: Ornamental-Artisanal, *M*: Medicinal, *WI*: Weather indicator, *C*: Ceremonial-Ritual, *AC*: Animal companion, *A*: Amulet, *T:* Tool, *CP*: Use for crop protection, *ME*: Melliferous

^b^Management categories: C/G: Manual capture and gathering, *H*: Hunting, *CC*: Care in captivity, *PG*: Planned gathering, *E*: Enhancing, *R*: Relocation, *T*: Trapping. Neither use or management categories are exclusive from one another.

We found evidence for treatments of at least twelve health issues, which involve animals (Table 3). Childbirth facilitation involves the use of 26.08% of the medicinal species, mainly mammals. Cancer treatment included 21.73% of the species, mainly snakes and birds. Treatments for skin problems and epilepsy both involved 17.39% of the species. Mammals, snakes and immature stages of Lepidoptera are used in the treatment of skin problems while mammals, snakes and birds are used for alleviating epileptic seizures. Trochilidae (hummingbirds) is the family used for more medical treatments. According to our ethnographic records in Coyomeapan, inhabitants of the region perceive that prior to the arrival of the vaccines in the 1970s, the use of birds and mammals to treat illnesses was much more common. An example of this is the larger former use of species of Trochilidae and Geomyidae Bonaparte, 1845 in remedies for whooping cough, especially for children.

**Table 3:** Healthcare and childbirth attention that involves animals, in the TCV and surrounding regions

Health issue	Cultural group	Animal Class	Animal Family	Number of species	Total number of species	Percentage of the total number of medicinal species (*n* = 23)
Giving birth	Nahua	Mammalia	Dasypodidae Gray, 1821, Didelphidae Gray, 1821, Tayassuidae Palmer, 1897, Erethizontidae Bonaparte, 1845, Cervidae Goldfuss, 1820	5	6	26.08
	Cuicatec	Mammalia	Dasypodidae, Didelphidae	2		
		Aves	Psittacidae Rafinesque, 1815	1		
Cancer	Nahua	Reptilia	Elapidae Boié, 1827, Viperidae Oppel, 1811	3	5	21.73
	Cuicatec	Reptilia	Viperidae	1		
	Mestizo	Aves	Cuculidae Leach, 1820, Corvidae Leach, 1820	2		
Dermatological	Nahua	Mammalia	Dasypodidae	1	4	17.39
		Insecta	Hepialidae Stephens, 1829	1		
		Reptilia	Elapidae	1		
	Cuicatec	Mammalia	Mephitidae Bonaparte, 1845	1		
Epilepsy	Cuicatec	Reptilia	Viperidae	1	4	17.39
		Mammalia	Mephitidae	1		
		Aves	Trochilidae Vigors, 1825	1		
	Mestizo	Aves	Cuculidae	1		
Antiophidic	Nahua	Reptilia	Elapidae, Viperidae	3	3	13.04
Whooping cough	Nahua	Mammalia	Geomyidae Bonaparte, 1845	1	2	8.69
		Aves	Trochilidae	1		
Hearth	Mestizo	Aves	Cuculidae, Trochilidae	2		
Cultural ilnesses	Cuicatec	Reptilia	Viperidae	1	2	8.69
	Mestizo	Aves	Trochilidae	1		
Joint pain/inflammation and rheumatism	Nahua	Mammalia	Canidae Fischer, 1817	1	1	4.34
	Ixcatec	Mammalia	Canidae	1		
Pain	Ixcatec	Insecta	Tenebrionidae Latreille, 1802	1	1	4.34
Antidepressant	Cuicatec	Insecta	Formicidae Latreille, 1802	1	1	4.34
Allergies	Cuicatec	Mammalia	Cervidae	1	1	4.34

Sources: [72, 73, 98, 101]; Rangel-Landa, Smith, Zarazúa and Chávez, this study

**Fig. 3:**
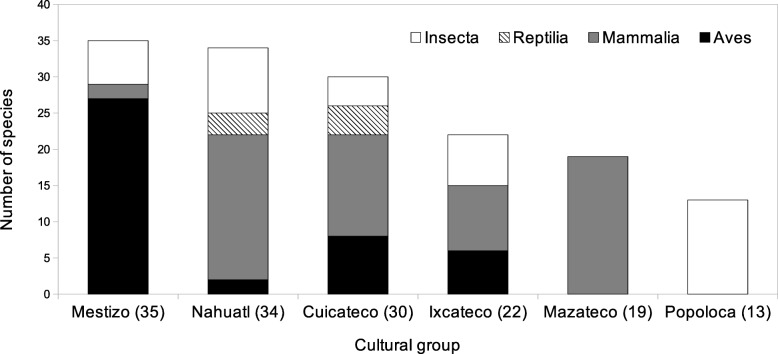
Use of animals by different cultural groups in the Tehuacán-Cuicatlán Valley and surrounding regions. Totals are indicated in parentheses

Each species can be used by 1 to 4 human groups (mean = 1.65 ± 0.09 SE, *n* = 93); 36 species are used by the Mestizo people, 32 by the Cuicatec and the Nahua, respectively, 23 by the Ixcatec, 19 by the Mazatec and 13 by the Popolocan people. The animals used by more cultural groups of the region are the mammals *Nasua narica* Linnaeus, 1766, *Pecari tajacu* Linnaeus, 1758, *Sciurus* spp. and *Sylvilagus* spp. (Fig. 3).

**Fig. 4:**
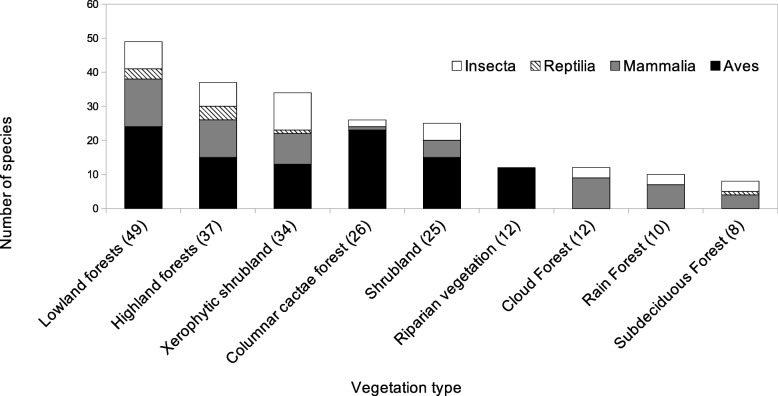
Occurrence of used fauna in different vegetation types in the Tehuacán-Cuicatlán Valley and surrounding regions. Totals are indicated in parentheses

The animals used in any of the categories documented have been reported in at least eight vegetation types along the TCV (Table 4, Fig. 4). This information should be considered as potential systems from which fauna is extracted, rather than the actual systems from which it is extracted.

**Table 4:** Occurrence of used fauna in different vegetation types in the TCV and surrounding regions

Vegetation type	Insecta	Reptilia	Aves	Mammalia	Total number of animal species	% of the total edible species (*n* = 74)	% of the total ornamental species (*n* = 30)	% of the total medicinal species (*n* = 23)
Lowland tropical dry forest	8	3	24	14	49	35.13	73.33	56.52
Highland temperate forest	7	4	15	11	37	33.78	43.33	47.82
Xerophytic shrubland	11	1	13	9	34	25.67	43.33	39.13
Columnar cacti forests	2	0	23	1	26	12.16	43.33	21.73
Shrubland	5	0	15	5	25	17.56	40	21.73
Cloud forest	3	0	0	9	12	16.21	16.66	21.73
Rain forest	3	0	0	7	10	13.51	13.33	13.04
Subdeciduous forest	3	1	0	4	10	8	3.33	21.73

### General management categories

**Fig. 5:**
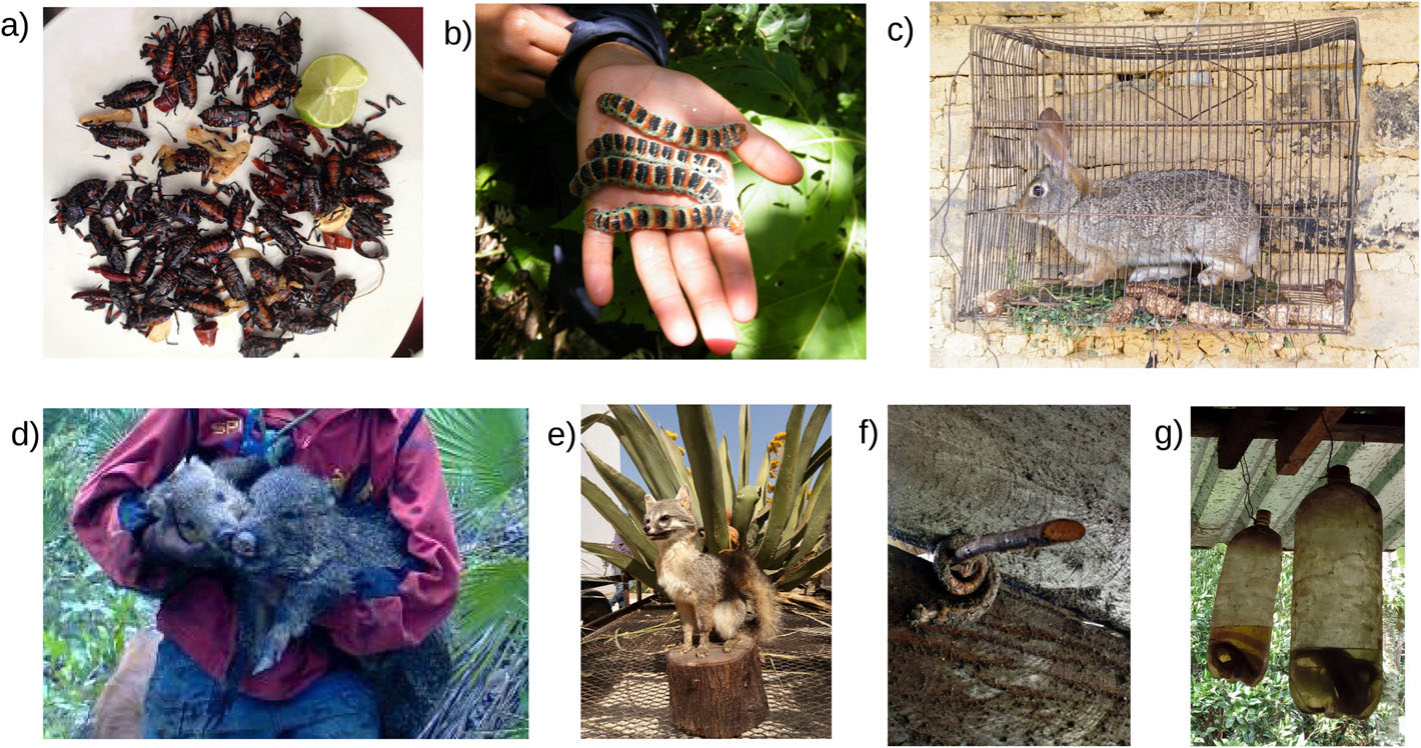
Examples of animals managed by people in the Tehuacán-Cuicatlán Valley and surrounding areas. **a** *Thassus gigas* (Hemiptera). The gathering of this edible insect from its host *Prosopis laevigata* involves planification, in contrast to the gathering of other insects that is performed in an opportunistic way, in a Popolocan locality. **b** *Arsenura armida* (Lepidoptera). Groups of this caterpillar “Cuetla” are gathered and then relocated in an *Heliocarpus aff. velutina* tree beside a family home, in Nahua localities. **c** *Sylvilagus* spp. (Lagomorpha). Juveniles are captured and maintained in captivity even for years, in Nahua localities. **d** *Pecari tajacu* (Artiodactyla). Juveniles are maintained in captivity in an Ixcatec locality. **e** *Urocyon cinereoargenteus* (Carnivora) is hunted and used as food in a Cuicatec locality. It is also considered to play pranks to pulque producers in the localities of Tehuacán. This picture shows one of them prepared with taxidermic techniques and exhibited in “La feria del Pulque”, Santa Ana Teloxtoc, 2019. **f** *Didelphis* spp. (Didelphimorphia) is hunted to prevent damages to domestic animals but also because of the use of its tail in childbirth in Nahua localities. **g** Snakes are captured alive and kept in alcohol to be used as antivenom, among other medical applications, in Nahua localities. Credits: **a**, **e**: SRL; **d**: FB; **b**, **c**, **f**, **g**: MZC

**Fig. 6:**
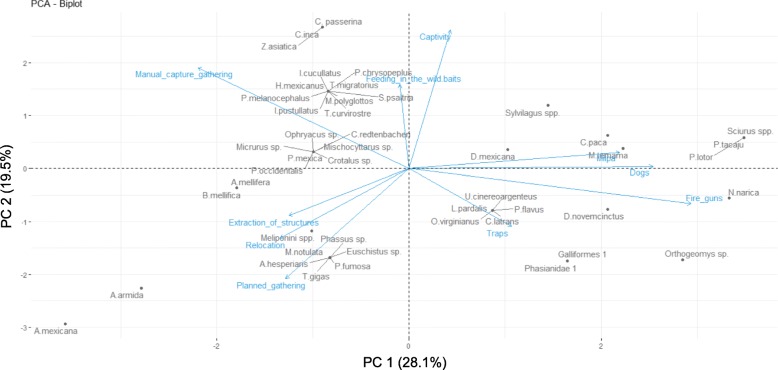
PCA biplot of the ordination of animal species on ten management variables. The first component (PC1) explains 28.10% of the variance and is mainly related to hunting, while the second (PC2) explains 19.50 % of the variance and is mainly related to captivity and different types of capture and gathering. Mammals in the upper right quadrant in the biplot are maintained in captivity, but also hunted in several ways. Mammals and Galliformes in the lower right quadrant of the biplot are captured using traps, but they can also be hunted. Insects and juvenile birds in the lower left quadrant in the biplot are manually collected, the extraction of some of them can involve regulated captures, extraction of nests or honeycombs, and relocation of individuals or structures

Animals in the TCV region are obtained through extractive practices. We recorded the following general management categories:

1. Hunting of vertebrates. it involves the chasing and/or killing of animals valued as resources with the use of fire guns, slingshots or projectiles such as stones (the latter specially in the case of birds). It might include the aid of hunting dogs or the use of baits. Hunting is performed individually or in groups of family members or friends. In some cases, it is a nocturnal activity which involves waiting for the animals to approach in the milpa during the maize production season, or in areas like “huertas” (a type of homegardens that includes portions of forest located next to homes, where desirable elements of the biotic community are maintained and from which plants, mushrooms and wild fauna can be extracted) or the forest, where the animals feed. For instance, the hunting of deer (*Odocoileus virginianus* Zimmermann, 1780 and *Mazama temama*) is likely a social activity that involves small groups of men and one or two dogs, while the hunting of *Cuniculus paca* Linnaeus, 1766 is an individual task that involves waiting at night for the animal in its feeding place, sometimes using baits. People can also go out searching for certain animals such as reptiles, mammals or birds, but there is also opportunistic killing when the animals are seen during a trip, as frequently occurs with *Didelphis* spp. Linnaeus, 1758, *Orthogeomys* sp. Merriam, 1895, or *Dasypus novemcinctus* Linnaeus, 1758*.* Hunting is also a way to reduce the damage caused by wild animals to crops or domestic animals, as is the case of *Orthogeomys* sp*.*, *Nasua narica* and *Pecari tajacu*, among others.
2. Trapping. in this category, we included the use of handmade cages or traps to capture animals. This is the case for two Galliformes Temminck, 1820 in Nahua localities in Coyomeapan. We included this practice because a few people still perform this kind of capture; however, people interviewed considered that this practice was much more frequent in the past.
3. Manual capture/gathering. Within this category, we grouped birds and mammal species that are manually collected from their habitat as juveniles or eggs (birds, *Sylvilagus* spp., *O. virginianus*, *M. temama*, *U. cinereoargenteus* (Schreber, 1975)*, P. tajacu*) and snakes which are captured alive with plastic bottles to prepare medicine (Fig. 5). We also included the non-regulated and non-planned gathering of insects. This is the case of the products of *A. mellifera*, Meliponini bees and wasp nests.
4. Relocation. It is a kind of gathering that involves collecting the animals and later transporting them to the desired place to increase their numbers, to facilitate their collection or that of their products, or to complete their development under surveillance. It is different from captivity since in relocation, people do not take active control of their feeding and movement. This is the case of the edible caterpillar *Arsenura* aff. a*rmida* (Cramer, 1779), which is gathered from *Heliocarpus aff. velutina* Rose, 1905 trees from the “acahual” and taken to an *Heliocarpus* tree in homegardens to be taken care of. A family might deposit as much as hundreds of these animals in the same tree to assure their availability either for direct consumption by households or to sell them in the regional markets (nearly 20 caterpillars per U.S. Dollar). *Atta mexicana* (Smith 1858) colonies are also relocated to desirable places because the soil they bring to the surface is valued as a plant fertiliser.
5. Planned gathering. It involves a planned and scheduled strategy to collect individuals or structures (i.e. bee and wasp combs, ant nests), sometimes also involving common agreements about gathering restrictions in time and/or space. It differs from other gathering actions that are more casual or opportunistic and non-regulated. This is the case of a few species of insects such as *Thasus gigas* (Klug in Burmester 1835), *Euschistus* sp. Dallas, 1851, *Mormidea notulata* (Herrich-Schäffer, 1844) (Hemiptera) and *Ormiscodes (Paradirphia) fumosa* Felder,1874 (Lepidoptera). It is also the case of the maguey worm *Aegiale hesperiaris* Walker, 1985 (Lepidoptera), whose recollection is restricted to a few days per year in some localities, to avoid overexploitation of this highly valued resource.
6. Enhancing. It refers to acts aimed to attract the animals by feeding them in the wild. It differs from relocation since the animals are not actively transported from one place to another but, rather, they are attracted to a certain location. This is the case of the birds *Columbina passerina* Linnaeus, 1758, *Columbina inca* Lesson, 1847 and *Zenaida macroura* (Linnaeus, 1758).
7. Care in captivity. It refers to activities to ex situ maintain animals, involving actions to control their feeding and restricting their movement capability. It does not involve the reproduction of the animal or the manipulation of its reproductive system. Animals that are maintained in captivity include the mammals *Sylvilagus* spp., *Sciurus* spp., *P. tajacu*, *Procyon lotor* (Linnaeus, 1758), *Dasyprocta mexicana* Saussure, 1860*,* juvenile individuals of *O. virginianus* and *M. temama* and ornamental birds. The individuals are often taken from their habitat as juveniles to be grown ex situ, in a family home. However, some of these animals often die in captivity after days or few weeks except for *Sylvilagus* spp., *Sciurus* spp. and birds, which can remain captive for years.
  These general categories are non-exclusive, and a single species can belong to several of them. An ordination of the species along ten variables of management actions within these general categories showed that the first principal component explains 28.10% of the variance and is mainly related to hunting, while the second principal explains 19.50% of the variance and is mainly related to captivity and different types of capture and gathering (Tables 5, 6; Fig. 6). Mammals like *Sylvilagus* spp., *Sciurus* spp. and *P. tajacu*, among others (upper right quadrant in the biplot) are maintained in captivity, but also hunted in several ways. Mammals like *Orthogeomys* sp., *U. cinereoargenteus*, *Canis latrans* Say, 1823, and species of Galliformes are or were captured using traps, but they can also be hunted (lower right quadrant in the biplot). Insects and juvenile birds are manually collected, and these can involve regulated captures, extraction of nests or honeycombs, and relocation of individuals or structures (lower left quadrant in the biplot).

**Table 5:** Results of the principal component analysis showing eigenvalues and explained variance for management actions

Component	Eigenvalue	Explained variance (%)	Cumulative explained variance (%)
PC1	2.81	28.10	28.10
PC2	1.95	19.50	47.60
PC3	1.29	12.92	60.53
PC4	1.03	10.33	70.86
PC5	0.91	9.19	80.05

**Table 6:** Scores of the management action variables in 5 principal components

Variable	PC1	PC2	PC3	PC4	PC5
Fire guns	0.88	−0.19	−0.03	0.10	−0.11
Milpa	0.66	0.09	0.56	−0.05	−0.13
Dogs	0.76	0.01	0.36	0.22	0.17
Traps	0.32	−0.32	−0.57	0.22	−0.32
Manual capture and gathering	−0.65	0.57	0.03	0.30	0.03
Feeding in the wild	−0.02	0.47	0.09	−0.38	−0.76
Captivity	0.12	0.78	0.20	0.03	0.18
Planned gathering	−0.38	-0.62	0.30	−0.49	0.11
Extraction of structures	−0.37	−0.26	0.27	0.63	−0.29
Relocation	−0.40	−0.39	0.53	0.15	−0.18

**Fig. 7:**
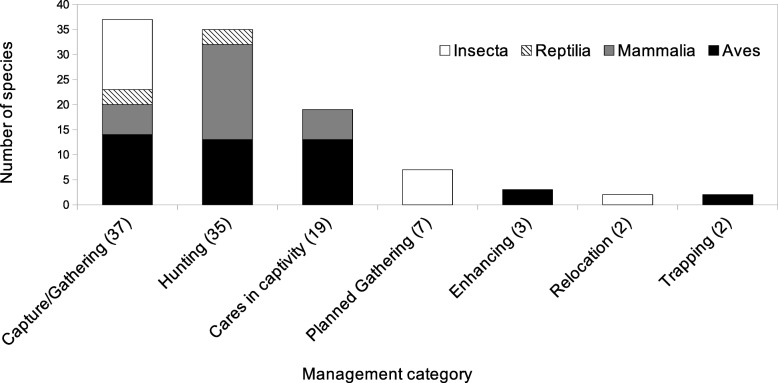
Management categories for animals in the Tehuacán-Cuicatlán Valley and surrounding regions. Totals are indicated in parentheses

The number of management categories per species ranges from 1 to 4 (all animals mean = 1.51 ± 0.08 SE, *n* = 71; birds mean = 1.76 ± 0.16 SE, *n* = 26; mammals mean = 1.61 ± 0.16 SE, *n* = 21; reptiles mean = 1 ± 0 SE, *n* = 6; insects mean = 1.27 ± 0.13 SE, *n* = 18) (Table 2, Fig. 7).

**Fig. 8:**
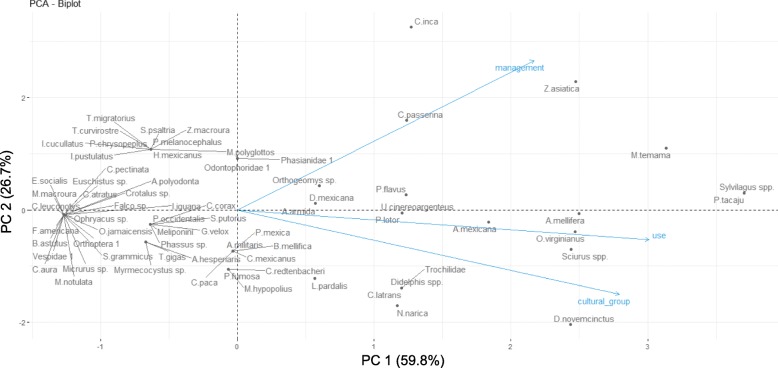
PCA biplot of the ordination of animal species on three variables: **1** number of use categories, **2** number of management categories, **3** number of cultural groups that use/manage the animal. The first principal component (PC1) explained 59.84% of the variance whereas the second one (PC2) explained 26.69% of it. Variation along the first principal component is mainly given by the differences in the number of uses and the number of cultural groups that use the species. Variation in the number of management categories was explained by both components. In the right half of the biplot, we find the species with more types of uses, more types of management and those which are used by more cultural groups. The species related with more types of management practices are concentrated in the upper right quadrant of the biplot

The ordination of species along with the number of uses, the number of management categories and the number of cultural groups suggested correlation among these three variables but this was only significant between the number of uses and cultural group (*r*_s_ = 0.586, *n* = 71, *p* value < 0.001). The first principal component explained 59.84% of the variance and the second one 26.69%. Most of the variation along the first principal component is given by the differences in the number of uses and the number of cultural groups that use the species. Variation in the number of management categories was explained by the first and the second principal components (Fig. 8, Table 7). In the right half of the biplot are the species with more types of uses, more types of management and which are used by more cultural groups. The species related to more types of management practices such as doves, the edible Lepidoptera larvae *A. armida*, the temazate deer (*M. temama*), cottontail rabbits (*Sylvilagus* spp.) and *P. tacaju* are concentrated in the upper right quadrant of the biplot.

**Table 7:** Results of the principal component analysis showing eigenvalues and explained variance for use, cultural group and management of animals

Component	Eigenvalue	Explained variance (%)	Cumulative explained variance (%)	Scores of variables^a^ on the principal component
PC1	1.79	59.84	59.84	Use: 0.86 Cultural group: 0.80 Management: 0.62
PC2	0.80	26.69	86.54	Use: −0.15217 Cultural group: -0.43 Management: 0.76
PC3	0.40	13.45	100.00	Use: −0.47158 Cultural group: 0.40 Management: 0.13

^a^Variables are the number of use categories for a given species, number of cultural groups that use a given species and number of management categories for a given species

### Animal use and management from 12,000 B.P. to 500 B.P.

Early settlers in the region integrated vertebrates and insects to their diet since before 12,000 B.P. Although the dates have been subject to discussion, the history of animal use in the TCV proposed by MacNeish, Flannery and Callen remains useful to characterise the general chronology of events. At least 20 of the species reported by these authors are still currently used in the area (Table 8).

**Table 8:** Edible fauna in archaeological evidence from 12,000 B.P. to 500 B.P. and its current type of use in the TCV

Animal	Time										Current use				
	12,000 BP–9000 BP	10,00BP–8700BP	8500BP–7000BP	7000BP–5500BP	5500BP–4300BP	4300BP–3500BP	3500BP–3000BP	3000BP–2200BP	2200BP–1300 PB	1300BP–500BP	Edible	Medicinal	Ornamental	Pets	Others
*Equuus* sp. Linnaeus, 1758^a^	x														
cf. *Antilocapra americana* (Ord, 1815)^a^	x														
*Lepus* sp. Linnaeus, 1758^a^	x			x											
Large fox^a^	x														
Small ground squirrel, chipmunk or prairie dog^a^	x														
*Gopherus* cf. *berlandieri* (Agassiz, 1857)^a^	x														
Quail^a^	x		x	x				x							
*Canis latrans* Say, 1823^b^	x			x			x				x				x
*Spilogale* sp.Gray, 1865^b^	x		x	x	x	x		x	x	x		x			x
*Mephitis* sp.É. Geoffroy Saint-Hilaire & F. G. Cuvier, 1795^b^	x								x	x		x			
*Bassariscus* sp.Coues, 1887^b^	x										x		x		
*Conepatus* sp.Gray, 1837^b^	x	x	x		x				x	x			x		
*Sylvilagus* spp.Gray, 1837^b^	x	x	x	x	x	x	x	x	x	x	x		x	x	x
*Odocoileus virginianus* Zimmermann, 1780 ^b^		x	x	x	x		x	x	x	x	x	x	x		
*Colinus virginianus* Linnaeus, 1758^b^		x									x				
*Pecari tajacu* Linnaeus, 1758^b^			x	x	x		x	x	x	x	x	x	x	x	x
*Urocyon cinereoargenteus* (Schreber, 1775)^b^			x	x	x				x	x	x		x		
*Didelphis* spp.Linnaeus, 1758^b^			x						x	x	x	x	x		
*Tyto alba* (Scopoli, 1769) ^b^				x					x		x		x		x
*Orthogeomys* sp.Merriam, 1895^b^				x				x		x	x	x			
*Procyon lotor* Linnaeus, 1758^b^					x			x		x	x		x		
*Zenaida asiatica* Linnaeus, 1758^b^			x	x	x				x				x		
*Columbina passerina* Linnaeus, 1758^b^			x	x	x				x		x		x		
*Iguana iguana* Linnaeus, 1758^b^								x	x	x	x				
*Ctenosaura pectinata* (Wiegmann, 1834)^b^								x	x		x				
*Corvus corax* Linnaeus, 1758^b^								x	x			x			x
*Falco* sp. Linnaeus, 1758^b^								x					x		
Fish^b^				x	x										x
Snakes^b^									x		x				
Birds^b^			x	x		x	x	x	x	x	x	x	x		
Insecta^b^			x	x	x		x			x	x	x			x
*Canis lupus familiaris* Linnaeus, 1758^c^					x		x	x	x	x					
*Meleagris gallopavo* Linnaeus, 1758^c^									x	x					
*Ictidomys mexicanus* (Erxleben, 1777) ^d^	x									x					
*Neotoma* spp. Say and Ord, 1825^d^	x														
*Peromyscus* spp.Gloger, 1841^d^	x														
*Liomys* sp.Merriam, 1902^d^	x														
*Puma concolor* Linnaeus, 1791^d^								x							
*Lynx rufus* (Schreber, 1777)^d^			x							x					
*Lepus* sp. 2^d^										x					
*Ameiva* sp. Meyer, 1795^d^		x	x	x	x		x	x	x	x					
*Kinosternon integrum* Le Conte, 1854^d^			x	x	x		x	x		x					

^a^Extirpated fauna

^b^Fauna with current use reports in the TCV and surrounding regions

^c^Incorporated domesticates

^d^Fauna without current use reports in the TCV and surrounding regions

According to the chronology reported, there is evidence of hunting and edible use of an American horse (*Equus* sp.) Linnaeus, 1758 and an antelope (Antilocapridae, probably *Antilocapra americana* (Ord, 1815)) about 12,000 B.P. along with small prey such as jackrabbits (*Lepus* sp. Linnaeus, 1758), coyotes (*C. latrans*), skunks (*Spilogale* sp. Gray, 1865, *Conepatus* sp. Gray, 1837), foxes (*Urocyon* sp. Baird, 1857), ring-tailed cats (*Bassariscus astutus* (Lichtenstein, 1830)), squirrels and turtles. Later, horses and antelope were extirpated from the area. From 10,000 B.P., the white-tailed deer (*O. virginianus*) appears in the archaeological record as hunted and consumed by humans, along with cottontails, lizards, skunks and other small prey. According to MacNeish, Flannery and Callen, small preys were probably obtained by trapping. Ants and immature stages of Lepidoptera were found in coprolites dated between 8500–7000 B.P. and 7000–5500 B.P., respectively, in the Coxcatlán area. Between 7000 and 4300 B.P., hunting of the white-tailed deer seems to have intensified, and there was also hunting of *P. tajacu* and trapping of small games. Around 5000 B.P., the domestic dog (*C. lupus familiaris*) was introduced into the area. Between 4300 and 3500 B.P., remains of dogs with evidences of human consumption increased. Between 3000 and 1800 B.P., gophers increased in the archaeological record. Then, around 1500 B.P., the first turkey (*M. gallopavo*) appeared in the area.

## Discussion

As we expected, fauna continues to be an important element of human culture and subsistence in the Tehuacán Valley, and the main use given to wild animal species, both vertebrates and invertebrates is for food. According to our field observations, this happens even in the presence of edible domesticates such as turkeys, chickens, pigs, goats and lambs.

The number of animal species used in the TCV as food, ornament and medicine is low compared with the number of plant species used for those purposes, but their proportions and importance are similar in these main use categories. Medicinal use of plants (396 species) is followed by edible (339 species) and ornamental (313 species) [4]. However, the fact that at least 874 plant species in the TCV are used as fodder [4], reflects the human interest in feeding animals in this region during the last five centuries.

Birds are the main taxonomic group that represents ornamental species, making this category the second in importance for edible purposes after mammals. This information comes mostly from the dry forests of the arid zone of the TCV [73]. Use and commercialization of living birds as ornamental is a complex activity that has a long tradition in Mexico and involves human specialization, organization and regulations at various levels from individuals and families, to non-profit associations and governmental policies [107]; however, with a few exceptions, breeding is not part of the management strategy for ornamental species [148]. So far, this is the case in the TCV.

The medicinal category was the third in the number of animal species and follows the tendency for Latin America, where the two groups with the larger numbers of medicinal species are mammals and birds [149]. We hypothesise that their uses can be taken as indicators of human health needs in the area. The presence of institutional medical attention could be resulting in the decrease in the use of animals to alleviate illnesses, as it has been reported for the use of medicinal plants in Ixcatlán [25]. In the Nahua localities of Coyomeapan, snakes are important components of the domestic first aid kit. They are used as antivenoms in a similar manner to that previously documented in localities of Brazil, where, in absence of antivenom treatments, coral snakes were introduced alive in brandy, and this brandy was then ingested to act as a preventive antivenom [150]. The alcohol used in the TCV is made of sugarcane, whose preparation was introduced after the Spanish conquest, and it is also used to preserve the tails or glands of mammals used in other treatments. An especially interesting situation is observed for childbirth attention. Frequently, infusions of animal tails or reproductive parts are used to induce nausea in women and thereafter, contractions. *Didelphis* spp. was the genus most widely used for this purpose in the region, even for animals’ birth. However, there is no consensus regarding its regional use since in some localities, other mammals such as *D. novemcinctus* and *Sphiggurus*  *mexicanus* (Ker, 1792) are used instead. *Didelphis* spp. fat is also used in childbirth in the Brazilian Amazon [151], but only to prepare an oil to alleviate labouring women’s pain.

We could argue that the study of the use and management of insects is still far from complete in the region, and the numbers do not reflect the general tendency registered for Mexico. We encourage more research on this subject in the area. Yet, a deep perspective of the uses and management of insects was documented in a case study of a Popolocan locality [97]. We also found a case of management of the immature stages of the lepidopteran *A. armida* in Nahua localities in Coyomeapan, where it is gathered but also receives a kind of in situ management that has been even called a “proto-culture” and has been documented in coffee agroecosystems in the Zongolica region [152]. The extent to which the interest for these caterpillars motivates special cares or management of the host tree (*H. aff. velutina*) beyond tolerance remains to be investigated.

Most of the obtained records of animal use do not include information about frequency and yields of the animals that are extracted, neither about the actual ecosystems from which the extracted animals came from. Contributions in these topics would be valuable together with basic ecological studies since human activity and extractive practices have caused the reduction and almost extirpation of big mammals such as deer and peccaries from some areas of the TCV [45, 47, 153].

Beyond the tangible-utilitarian attributes reported in the results of this work, the presence of certain animals in an area also may acquire an intangible dimension of use that, in turn, can lead to concrete management actions with positive or negative results for animal survival. For example, fauna which represents bad omens (*Catharthes aura* (Linnaeus, 1758), *Coragyps atratus* (Bechstein, 1793), *Geococcyx velox* (Wagner, 1836), *Glaucidium brasilianum* (J.F. Gmelin, 1788)) or good fortune (*Pyrocephalus rubinus* (Boddaert, 1783), *Toxostoma curvirostre* (Swainson,1827)*, Cynanthus sordidus* (Gould, 1959)) [72, 98]. This might also be the case of fauna mistakenly considered to be dangerous for human health, as it happens with lizards of the genus *Abronia* spp. Gray, 1838 Another example is the use of hummingbirds as amulets; this might increase the risk of extirpation since no strategies for maintaining or restoring their populations was recorded. We recommend further research on this topic.

The management typology found so far in the TCV includes a variety of extractive practices similar to those documented in other regions of Mesoamerica. However, we have not found any evidence of breeding as it has been documented for some animals in other regions of Mesoamerica, as it is the case of stingless bees in the Maya region and in the Sierra Norte in Puebla, among others [15, 154]. We did not find evidence of the manipulation of abiotic elements with the purpose of enhancing the availability of animals [5], nor manipulation at community level with the only purpose of attracting animals as reported in the Maya region [3]. Future research is needed to determine if the documented practices aimed at increasing the availability of the desirable animals are likely to be successful; therefore, we recommend investigating the outcome of these practices.

The use and management of fauna before 1500 B.P. has been discussed in the context of the history of the origins of agriculture in the region [46–48] and involves a transition from seasonal hunting in great human groups to small groups. It might also be related to irrigated cultivation systems by allowing hunters to capture prey attracted to the systems, similar to what happens currently with the milpa. Captivity or breeding of wild animals are not considered to have happened in the region before 1500 B.P., except for *D. coccus* [155] and the possibly introduced wild *M. gallopavo* around 2200 B.P. However, *Sylvilagus* spp. were found in very high proportions compared to other preys and it has been hypothesised that it was due to massive hunting/trapping events [47]. It could be possible that cottontails were captured and maintained in captivity, as it currently happens in some localities of the region, and as it was registered to have possibly happened in Teotihuacan [18].

Up to 2010, there was documentation of 10 animal species found in the archaeological record which are still currently used [153]. Through the review of information carried out in this study, we found reports for 10 additional species which continue to be used, making a total of 20 species. Although in the last 8 years studies on the use and management of regional fauna have increased, we have identified a lack of information on the subject for the Mixtec, Chinantec and Chocholtec communities of the TCV.

Epistemological discussion in ethnobiology and ethnozoology is dynamic [156]. Trends in the thematic of ethnozoological research in Mexico have been reviewed and classified by Brand (1962), Argueta et al. (2012), Santos-Fita et al. (2012) and Gutiérrez-Santillán et al. (2019) [156–158] among others. Works about extractive practices and use of fauna with feeding and ornamental purposes, as well as management issues, specifically domestication or taming, have been present in ethnozoological studies in Mexico. For the TCV, one of the first archaeological studies in which authors discuss these aspects was published in 1967, but the frequency of studies related to management has increased recently. For the period 1890–1962, Brand (1962) identified 4 studies related to hunting and the importance of animals in human diet, 7 studies on domestication and semi-domestication, 15 studies on ornamental use. For the period 1962–2001, Argueta et al. 2012 identified 26 studies on hunting, harvesting, importance in human diet and commercialization, 24 related to use as ornamental, tools, building and dressing elements and 18 studies related to animals in traditional medicine. For the period 2000 to 2011, Santos-Fita et al. 2012 identified 75 studies on hunting, harvesting, diet and commercialization, 16 studies on ornamental use, 30 about animals in traditional medicine and 26 on management and domestication.

A limitation of our work was the lack of analyses on the incorporation of invertebrate and vertebrate domesticates in local systems of subsistence. Further studies on these subjects would complement our understanding of the importance of wild animal resources to fulfil people’s needs, as well as the ecological and social processes that might emerge from the management of fauna in the region. An example of work that evaluates the interactions between introduced domesticates in the TCV and vegetation are the studies that have characterised the plant species consumed by goats [159, 160] and those that have assessed the effects of goat herbivory on the growth and flower set of plants [161]. Although goats have been considered detrimental for ecosystems, several authors have documented that, at the right densities, these animals may contribute to the conservation of plant diversity and participate in seed dispersal of numerous species [162–164]. Other important issues are the local criteria for the human selection of domestic animals. Adaptation to local environments and cultural motivations are important issues to characterise local genetic resources and to complete a history of animal use and management in a region where agriculture and domestication had one of its earliest origins.

## Conclusions

Wild animals are still valuable resources for the inhabitants of the TCV to satisfy mainly edible, ornamental and medicinal needs. To obtain these animals, people in the area perform extractive practices including hunting, gathering and care in captivity among others. Such practices, to our knowledge, do not involve active processes of artificial selection that lead to domestication. However, because of the variation among the strategies involved, we have discussed a typology of extractive practices in the area. Further studies on the qualitative description of these practices, the reasons that have motivated humans to choose them, and the outcome of these practices on animal populations might help us to understand the origins of animal management scenarios and to contribute to biodiversity conservation schemes. It seems to us that ethnozoological information is still lacking in the area since 178 species present in the area without local use reports are used in other regions of Mesoamerica. Besides, ethnozoological information is still not reported among the Mixtec, Chinantec and Chocholtec people of the region.

## Supplementary information

**Additional file 1..** VTC localities. Checklist of the localities within the study area.

**Additional file 2..** Documentary research conceptual map. Image of a conceptual map in which the documentary research process, including keywords, is summarized.

**Additional file 3..** Synthesis of the list of used and managed species in the Tehuacan-Cuicatlan area and surrounding regions used for the analyses. Tabular data with taxonomic information of animals to species level, indicating use and management categories within TCV and its references. Information for each species is summarized in a single line.

**Additional file 4..** Records of used and managed species in the Tehuacan-Cuicatlan area and surrounding regions. Tabular data with taxonomic information of animals to species level indicating use and management categories and references.

**Additional file 5..** List of animals present in the Tehuacan-Cuicatlan area and surrounding regions, but with use reports in other regions of Mesoamerica. Tabular data with taxonomic information of animals to species level and references. Each line is a species and at least one reference of its presence within the study area in the TCV, and one reference of its use in other regions of Mesoamerica.

## Data Availability

The datasets used and/or analysed during the current study are available from Additional files 3, 4 and 5.

## Abbreviations

CIByC: Centro de Investigación en Biodiversidad y Conservación
CONABIO: Comisión Nacional para el Conocimiento y Uso de la Biodiversidad
CONACYT: Consejo Nacional de Ciencia y Tecnología
DGAPA: Dirección General de Apoyo al Personal Académico
ENES: Escuela Nacional de Estudios Superiores-Morelia
FES-Iztacala: Facultad de Estudios Superiores-Iztacala
GBIF: Global Biodiversity Information Facility
IIES: Instituto de Investigaciones en Ecosistemas y Sustentabilidad
ITIS: Integrated Taxonomic Information System
PAPIIT: Programa de Apoyo a Proyectos de Investigación e Innovación Tecnológica
TCV: Tehuacan-Cuicatlan Valley
UNAM: Universidad Nacional Autónoma de México
