# Supplementary figures and images for: Use and management of wild fauna by people of the Tehuacán-Cuicatlán Valley and surrounding areas, Mexico
Source: J Ethnobiol Ethnomed. 2020 Jan 28;16:4. doi: 10.1186/s13002-020-0354-8 (PMC6986097; doi:10.1186/s13002-020-0354-8)

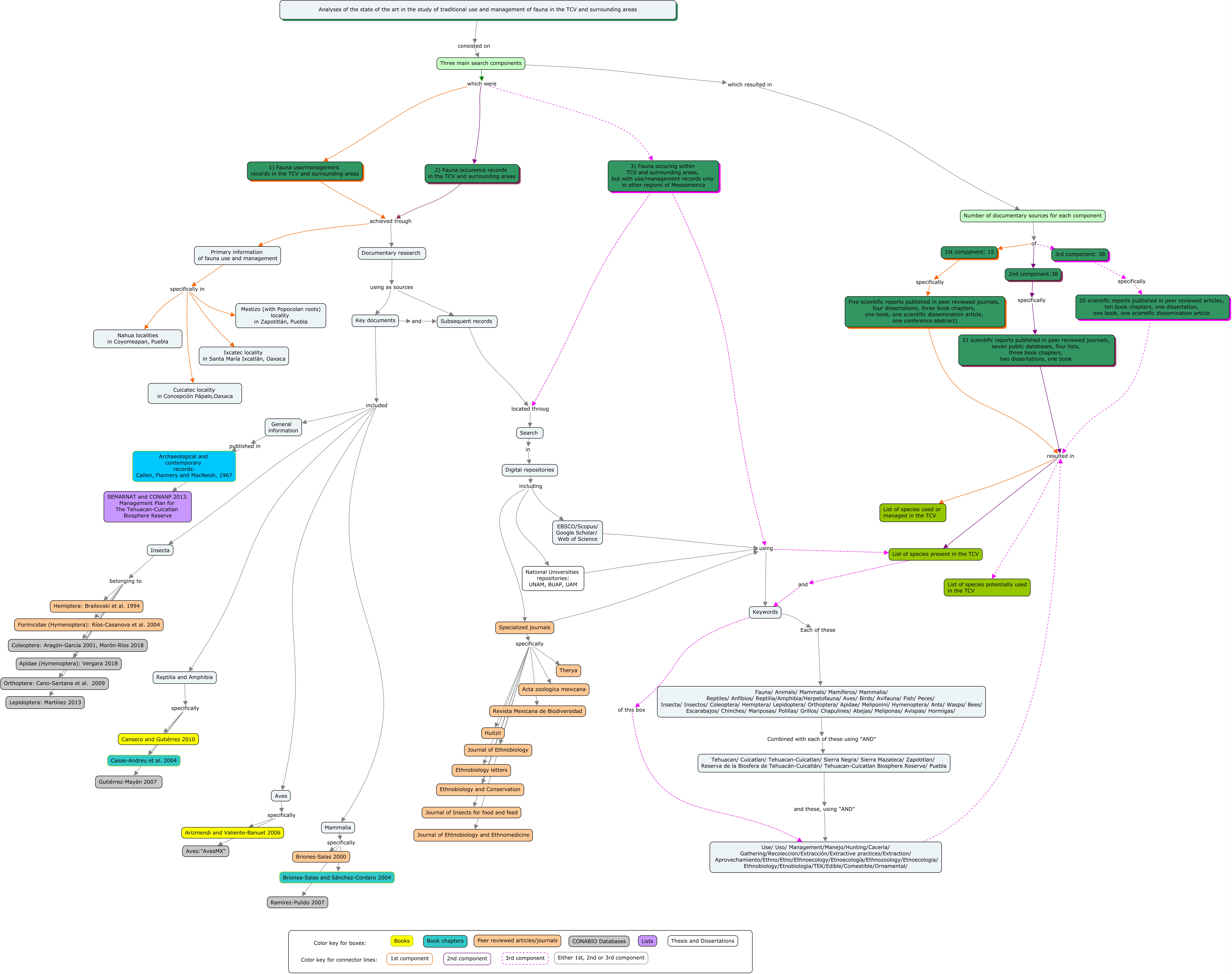

Supplement: Supplementary file 2 — Additional file 2.. Documentary research conceptual map. Image of a conceptual map in which the documentary research process, including keywords, is summarized. [file 13002_2020_354_MOESM2_ESM.jpg]
